# Divergent soil P accrual in ectomycorrhizal and arbuscular mycorrhizal trees: insights from a common garden experiment in subtropical China

**DOI:** 10.3389/fpls.2024.1333505

**Published:** 2024-02-07

**Authors:** Pingping Lian, Linglin Xu, Liuming Yang, Kai Yue, Josep Peñuelas

**Affiliations:** ^1^ School of Geographical Science, Fujian Normal University, Fuzhou, China; ^2^ State Key Laboratory of Subtropical Mountain Ecology (Funded by Ministry of Science and Technology and Fujian Province), Fujian Normal University, Fuzhou, China; ^3^ School of Design, Fujian University of Technology, Fuzhou, China; ^4^ Institute of Geography, Fujian Normal University, Fuzhou, China; ^5^ Ecological and Forestry Applications Research Center (CREAF), Cerdanyola del Vallès, Spain; ^6^ CSIC, Global Ecology Unit CREAF-CSIC-UAB, Cerdanyola del Vallès, Spain

**Keywords:** organic P, enzyme activity, foliar P, foliar Mn, foliar nutrient stoichiometry, the PLFAs

## Abstract

Tree species establish mycorrhizal associations with both ectomycorrhizal (EM) and arbuscular mycorrhizal fungi (AM), which play crucial roles in facilitating plant phosphorus (P) acquisition. However, little attention has been given to the effects of EM and AM species on soil P dynamics and the underlying mechanisms in subtropical forests, where P availability is typically low. To address this knowledge gap, we selected two EM species (*Pinus massoniana* - PM and *Castanopsis carlesii* - CC) and two AM species (*Cunninghamia lanceolata* - Chinese fir, CF and *Michelia macclurei* - MM) in a common garden established in 2012 in subtropical China. We investigated soil properties (e.g., pH, soil organic carbon, total nitrogen, and dissolved organic nitrogen), soil P fractions, phospholipid fatty acids (PLFAs), enzyme activities, foliar manganese (Mn) concentration, and foliar nutrients and stoichiometry. Our findings revealed that soils hosting EM species had higher levels of resin P, NaHCO_3_-Pi, extractable Po, total P, and a greater percentage of extractable Po to total P compared to soils with AM species. These results indicate that EM species enhance soil P availability and organic P accumulation in contrast to AM species. Moreover, EM species exhibited higher P return to soil (indicated by higher foliar P concentrations) when compared to AM species, which partly explains higher P accumulation in soils with EM species. Additionally, resin P showed a positive correlation with acid phosphatase (ACP) activity, whereas no correlation was found with foliar Mn concentration, which serves as a proxy for the mobilization of sorbed soil P. Such findings indicate that organic P mineralization has a more substantial impact than inorganic P desorption in influencing P availability in soils hosting both EM and AM species. In summary, our study contributes to a more comprehensive understanding of the effects of mycorrhizal associations on soil P accumulation in subtropical forests and provide valuable insights into plant-soil interactions and their role in P cycling in regions with limited P availability.

## 1 Introduction

Phosphorus (P) is a vital nutrient for plant growth, playing a crucial role in sustaining terrestrial ecosystems’ productivity and functionality, especially in tropical and subtropical forest soils where P is often strongly fixed and has low availability (Vitousek et al., 2010; Hidaka and Kitayama, 2011; Mirriam et al., 2022; Suriyagoda et al., 2023). To combat nutrient deficiencies, plants have formed symbiotic relationships with soil microorganisms, particularly mycorrhizal fungi (Phillips et al., 2013; Tedersoo et al., 2020; Li et al., 2022). Specifically, tree species establish associations with two main types of mycorrhizal fungi, ectomycorrhizal (EM) and arbuscular mycorrhizal fungi (AM), which significantly enhance the efficient acquisition of soil P (Landeweert et al., 2001; Seleiman et al., 2013; Rosling et al., 2016; Seleiman and Hardan, 2021; Wang et al., 2023). However, the extent to which EM and AM species influence soil P dynamics and the underlying mechanisms remain insufficiently understood.

Soil P exists in complex forms, primarily as organic P and inorganic P (Hedley et al., 1982; Seleiman et al., 2020; Cheptoek et al., 2021). Organic P cannot be directly assimilated by plants, while most inorganic P is absorbed by soil minerals, resulting in the majority of soil P being non-bioavailable (Tiessen and Moir, 1993; Seleiman, 2014). When faced with P limitations, plants may increase phosphatase activity to mineralize organic P or release carboxylates to liberate sorbed inorganic P (Plassard et al., 2019; Zhang et al., 2023). Recent studies have demonstrated that the leaf Mn concentration is often positively related with rhizosphere carboxylates, and can be used as a useful proxy that reflects the mobilization of sorbed soil inorganic P (Lambers et al., 2015; Lambers, 2022; Yu et al., 2023a). However, the efficiency of mobilizing recalcitrant P in soil can vary depending on the type of mycorrhizal association formed by the plant (Tedersoo and Bahram, 2019; Jiang et al., 2022). AMF species are good at activating sorbed inorganic P, while EMF species are effective in hydrolyzing organic P, which potentially affecting the composition and availability of soil P. Consequently, this variability might explain the inconsistent response of soil P fractions to changes in environmental conditions.

EM and AM species have evolved the ability to convert unavailable P into bioavailable forms through organic P mineralization and inorganic P desorption (Richardson et al., 2011; Jiang et al., 2021). Previous studies have indicated that EM species generally exhibit greater enzymatic capability for hydrolyzing organic P compared to AM species (Read and Perez-Moreno, 2003; Phillips et al., 2013). This observation is supported by studies in temperate forests, which have shown lower organic P levels and higher acid phosphatase (ACP) activity in soils dominated by EM species (Rosling et al., 2016). However, contrasting results were found in a study conducted in a subtropical karst forest, where lower phosphatase activity and reduced available P were observed in soils dominated by EM species when compared to those dominated by AM species (Yang et al., 2021). These findings highlight that the type of mycorrhizal association in trees plays a role in shaping P forms in soil, although the underlying mechanisms are not yet fully understood.

Variations in P demand among different tree species could be an additional factor contributing to differences in soil P status (Weand et al., 2010; Guilbeault-Mayers et al., 2020; Lu et al., 2023; Wagenknecht et al., 2023; Zheng et al., 2023). Previous studies have found that soil microbes account for 68-78% of the total biomass P in mature forests, but only 20% of them in 5-year-old forests (Turner et al., 2013). These findings suggest that plant P, such as foliar P, has a lesser impact on the soil P pool in mature forests but may significantly influence soil P dynamics in young forests. Therefore, young trees with rapid growth and high P demand (Chen et al., 2023), as indicated by foliar C:P ratios, might extract more P from the soil, resulting in lower soil P levels. Additionally, tree species with high foliar P concentrations might return more P to the soil through litterfall, which could be advantageous for soil P accumulation (González et al., 2022). Generally, EM species tend to adopt a conservative nutrient strategy and exhibit relatively lower growth rates, while AM species employ an acquisition strategy and display rapid growth. This distinction implies that AM species may have higher P demands compared to EM species, potentially hindering P accumulation in soils where AM species dominate. However, it is important to note that further validation is required to support this assertion.

To investigate the impact of trees with different mycorrhizal types on the availability and accumulation of P in subtropical forest soils with P limitations, we selected four tree species, including two EM species, *Pinus massoniana* (PM) and *Castanopsis carlesii* (CC) (Fan et al., 2018; Pang et al., 2023; Yang et al., 2023), and two AM species, *Cunninghamia lanceolata* (Chinese fir, CF) and *Michelia macclurei* (MM) (Cui et al., 2019; Li L. et al., 2019; Ma et al., 2023). We conducted investigations into soil properties, soil P fractions, phospholipid fatty acids (PLFAs), enzyme activities, foliar manganese (Mn) concentration, and foliar nutrients and stoichiometry. Our objectives were to: 1) examine changes in soil P fractions after planting EM or AM species following an 8-year cultivation period; 2) compare foliar P content and nutrient stoichiometry between EM and AM species; and 3) identify the key factor predominantly influencing soil P status and availability in different mycorrhizal species.

## 2 Methods and materials

### 2.1 Study site

The study site is located within the Sanming Forest Ecosystem and Global Change National Observatory and Research Station, situated in Fujian Province, China (26°11′N, 117°228′E). This region experiences a subtropical monsoon climate, characterized by an average annual temperature of 19.5 °C. The average annual precipitation measures approximately 1656 mm, with 77% of this total rainfall occurring between March and August. The evaporation rate is around 1585 mm. The soil is classified as an Oxisol, derived from sandstone, and is categorized as Fluventic Dystrochrept in the USDA soil classification system (Fan et al., 2020).

### 2.2 Experimental design

To investigate the influence of different mycorrhizal tree species on soil nutrient turnover and the underlying mechanisms, a common garden experiment was established in February 2012. The experiment comprised four tree species: two EM species (CC and PM) and two AM species (CF and MM). Following clearcut logging and burning in a secondary forest, 2-year-old saplings were planted at a density of 2,860 seedlings per hectare. A total of 16 plots were arranged in a random block design, each plot was planted a single tree species and covering an area of approximately 0.4 hectares. Each tree species was replicated 4 times. Additionally, a 2-meter buffer zone was implemented between plots to minimize mutual interference.

### 2.3 Soil and leaf sampling

Soil sampling was conducted in July 2020. Within each plot, 10 cores were randomly collected from 0-10 cm depth using a stainless-steel sampler with a diameter of 5 cm. After removing stones, roots, and plant and animal residues, the soil cores were combined to create a composite sample, which was then sealed in a plastic bag. A total of 16 samples (4 tree species × 4 replications) were transported to the laboratory, where samples were sieved through a 2 mm mesh and divided into three separate subsamples. The first subsample was stored at -20°C for the analysis of enzyme activity and phospholipid fatty acid (PLFA). The second subsample was air-dried and used for determining soil pH, soil organic carbon (SOC), total nitrogen (TN), and soil P fraction. Finally, the third subsample was stored at -4 °C and used to detect NH_4_^+^-N, NO_3_^–^N, and dissolved organic nitrogen (DON).

In each plot, 5 trees with an average diameter at breast height were selected, 200 g of mature leaves in different direction of each tree were collected, and approximately 1000 g of leaves were sampled in each plot. A total of 16 leaf samples (4 tree species × 4 replicates) were brought to the laboratory for processing. The leaves samples were heated at 105°C for 30 minutes to eliminate any living organisms. They were then dried at 65°C for 48 hours and subsequently ground in a ball mill. The resulting ground leaf samples were stored in polypropylene vials for the determination of C, N, P, and manganese (Mn) concentrations.

### 2.4 Soil and leaf nutrient analysis

SOC and TN were quantified using a C-N analyzer (ElementarVario, MAX, Germany). Soil pH was measured using a pH meter in a 1:2.5 soil-water suspension. DON was extracted by mixing the soil with deionized water in a 1:4 ratio, then analyzed using a Continuous Flow Analytic System (SAN++; Skalar, Netherlands). NH_4_^+^-N and NO_3_^–^N were extracted using a 2 M KCl solution, then analyzed using the Continuous Flow Analytic System (SAN++; Skalar, Netherlands).

Soil P fractions were assessed using a sequential extraction method based on Hedley et al. (1982) and Tiessen and Moir (1993). Briefly, 0.5 g of air-dried soil was sequentially extracted by deionized water and one resin strip, 0.5 M NaHCO_3_, 0.5 M NaOH, 1M HCl, and subsequent digestion of the residual fraction using H_2_SO_4_-H_2_O_2_ at 360 °C. The P concentration in each extract was measured using a Continuous Flow Analytic System (SAN++; Skalar, Netherlands). The NaHCO_3_, NaOH, and HCl extracts contained both inorganic P (Pi) and organic P (Po). Pi was the P concentration in these extracts without digestion, and Po was the difference between the total P digested and the Pi in each extract. Soil P fractions included resin P, NaHCO_3_-Pi, NaHCO_3_-Po, NaOH-Pi, NaOH-Po, HCl-Pi, HCl-Po, and residual-P. Extractable Po was the sum of NaHCO_3_-Po, NaOH-Po, and HCl-Po, while extractable Pi was the sum of NaOH-Pi and HCl-Pi. Total P was the sum of all P fractions.

Foliar C and N concentrations were measured using the same C-N analyzer (ElementarVario, MAX, Germany). Foliar P and Mn concentrations were determined using inductively coupled plasma-mass spectrometry after digesting the leaf samples with HNO_3_-HClO_4_.

### 2.5 PLFA analysis

PLFA analysis was conducted following a previously established protocol. In brief, soil samples were extracted using a phosphate buffer in the Bligh-Dyer extraction method. The resulting extraction solution was then purified using solid-phase extraction. The extracted compounds were further transesterified to generate fatty acid methyl esters, which were subsequently analyzed using gas chromatography equipped with a flame ionization detector. Fatty acids were identified utilizing the PLFAD1 method in Sherlock software (MIDI Inc., Newark, DE, USA) and quantified using the internal standard 19:0. The assignment of specific phospholipid fatty acids to microbial groups was based on (Swallow et al., 2009; Frostegård et al., 2011; Fan et al., 2020), the PLFAs i14:0, i15:0, a15:0, i16:0, a17:0, and i17:0, as well as 16:1ω7c, cy17:0, 18:1ω7, and cy19:0, were designated as bacterial biomarkers. The PLFAs 18:1ω9c and 18:2ω6,9c was used as fungal biomarker.

### 2.6 Enzyme activity assay

The activity of 4-N-acetylglucosaminidase (NAG), acid phosphatase (ACP), beta glucosidase (βG), and cellobiohydrolase (CBH) was assessed following the experimental procedure described by Saiya-Cork et al. (2002). Briefly, fresh soil samples weighing 1 g were extracted using 125 mL of acetate buffer. The soil samples and buffer were homogenized using a Brinkmann Polytron PT 3000 homogenizer, resulting in soil suspensions. To initiate the enzyme assay, 200 μL of the soil suspensions and 50 μL of fluorescence enzyme substrate (βG, 4-methylumbelliferyl-β-D-glucoside; NAG, 4-methylumbelliferyl-N-acetyl-β-D-glucosaminide; CBH, 4-methylumbelliferyl-β-D-cellobioside; ACP, 4- methylumbelliferyl -phosphate) were added to 96-well microplates, with each sample having 16 replicate wells. The microplates were then incubated at a temperature of 20°C for a duration of 4 hours, in the absence of light. The enzyme activity was subsequently determined using a Multifunction microplate reader with 365 nm excitation and 450 nm emission filters (Synergy H4, America).

### 2.7 Statistical analysis

Statistical analyses were conducted using IBM SPSS Statistics 21, version 19.0 (IBM, Armonk, NY, USA), and graphical representations were generated using Origin 9.0 software (Origin Lab, Massachusetts, USA). One-way analysis of variance (ANOVA) was employed to determine the significance of differences among the four forest plantations in terms of soil properties, leaf nutrition, soil P fractions, PLFAs, and enzyme activity. The distinction between arbuscular mycorrhizal tree species and two ectomycorrhizal tree species was investigated via a samples t-test. The study also utilized the random forests algorithm via the “random Forest” package in the R platform to assess the predictive capacity of soil properties, leaf nutrition, microbial biomass, and soil enzyme activity regarding available P (Resin P) and organic P (extractable Po). Any P-value less than 0.05 was considered significant in this study.

## 3 Results

### 3.1 Soil properties

There were no statistically significant differences in pH, SOC, TN, DON, and NH_4_^+^-N between EM and AM species soils (*P* > 0.05), except for higher NO_3_^–^N levels in soils of EM species compared to AM species (*P* < 0.001, **Table 1**). Similarly, no significant differences in pH, SOC, TN, and NH_4_^+^-N were observed among four forest soils (*P* > 0.05, **Table 1**). However, the concentration of DON in MM was higher than in PM, CC, and CF (*P* < 0.05), and the NO_3_^–^N levels in PM and CC were higher than those in CF and MM (*P* < 0.05).

**Table 1:** Soil properties in *Pinus massoniana* (PM), *Castanopsis carlesii* (CC), Chinese fir (CF), and *Michelia macclurei* (MM) forest soils in subtropical China.

Soil properties	EM species		AM species		*t*	*P*
	PM	CC	CF	MM		
pH	4.46 ± 0.18a	4.48 ± 0.06a	4.42 ± 0.13a	4.38 ± 0.16a	1.055	0.309
SOC (g kg^-1^)	17.17 ± 2.97ab	16.71 ± 0.57ab	15.42 ± 1.36b	18.45 ± 1.56a	-0.001	0.999
TN (g kg^-1^)	1.28 ± 0.08a	1.31 ± 0.08a	1.20 ± 0.12a	1.33 ± 0.08a	0.769	0.457
DON (mg kg^-1^)	3.00 ± 0.91b	3.01 ± 0.40b	3.01 ± 1.77b	6.24 ± 1.55a	-1.911	0.092
NH_4_^+^-N (mg kg^-1^)	9.44 ± 1.78a	11.18 ± 2.95a	8.62 ± 0.81a	8.36 ± 0.97a	1.993	0.079
NO_3_^–^N (mg kg^-1^)	0.40 ± 0.10a	0.35 ± 0.11a	0.12 ± 0.03b	0.11 ± 0.02b	7.144	< 0.001***

EM species, ectomycorrhizal species; AM species, arbuscular mycorrhizal species; SOC, soil organic carbon; TN, total nitrogen; DON, dissolved organic nitrogen. Different letter presents statistical difference among four species (P < 0.05); *** present statistical difference (P < 0.001) between EM and AM species.

### 3.2 Soil P fractions

**Figure 1:**
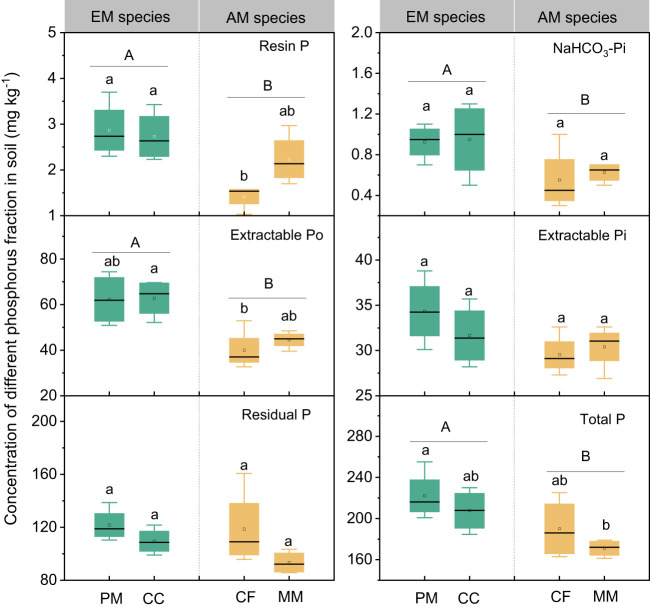
Soil phosphorus fractions in *Pinus massoniana* (PM), *Castanopsis carlesii* (CC), Chinese fir (CF), and *Michelia macclurei* (MM) forests in subtropical China. EM species, ectomycorrhizal species; AM species, arbuscular mycorrhizal species. Different lowercase letter presents statistical difference among four species (*P* < 0.05). Different uppercase letter presents statistical difference between EM and AM species (*P* < 0.05).

**Figure 2:**
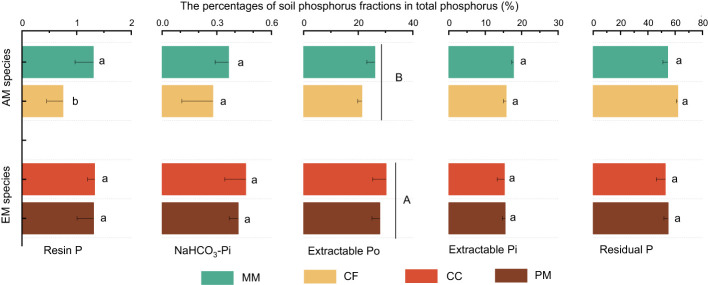
The percentage of different phosphorus fractions in total phosphorus at *Pinus massoniana* (PM), *Castanopsis carlesii* (CC), Chinese fir (CF), and *Michelia macclurei* (MM) forests in subtropical China. EM species, ectomycorrhizal species; AM species, arbuscular mycorrhizal species. Different lowercase letter presents statistical difference among four species (*P* < 0.05). Different uppercase letter presents statistical difference between EM and AM species (*P* < 0.05).

The influence of tree species on soil P varied with different P fractions (**Figure 1**). Resin P and extractable Po were lower in CF than in PM, CC, and MM, while the total P was higher in PM than in MM. There were no significant differences in NaHCO_3_-Pi, extractable Pi, and residual P among the four forest soils (*P* > 0.05, **Figure 1**). Moreover, mycorrhizal type significantly influenced soil P fractions, with resin P, NaHCO_3_-Pi, extractable Po, and total P being higher in EM species soils than in AM species forest soils (*P* < 0.05, **Figure 1**). Regarding the proportion of different P fractions in total P, neither tree species nor mycorrhizal type had a significant effect, except that the extractable Po in EM species was higher than in AM species soils (*P* < 0.05, **Figure 2**).

### 3.3 Leaf nutrient stoichiometry and Mn concentration

The foliar C, N, and P concentrations in PM and CC were higher than in CF and MM species (*P* < 0.05), while the values of foliar C:P and C:N ratios in PM and CC were lower than in CF and MM species (*P* < 0.05, **Table 2**). Compared with AM species, foliar N and P were higher but the foliar C:P and C:N were lower in EM species (*P* < 0.05, **Table 2**). There were no significant effects of mycorrhizal type on foliar C and foliar N:P (*P* > 0.05, **Table 2**). Additionally, neither tree species nor mycorrhizal type had significant influences on foliar Mn concentration (*P* > 0.05, **Table 2**).

**Table 2:** Leaf nutrient stoichiometry of *Pinus massoniana* (PM), *Castanopsis carlesii* (CC), Chinese fir (CF), and *Michelia macclurei* (MM) forest in subtropical China.

Foliar nutrient	EM species		AM species		*t*	*P*
	PM	CC	CF	MM		
Foliar C (g kg^-1^)	501.17 ± 7.83a	472.88 ± 2.69c	483.01 ± 6.43b	470.45 ± 1.37c	1.624	0.135
Foliar N (g kg^-1^)	15.47 ± 1.04b	18.42 ± 0.16a	10.22 ± 1.24c	12.13 ± 2.75c	5.815	< 0.001**
Foliar P (g kg^-1^)	1.03 ± 0.05b	1.50 ± 0.14a	0.85 ± 0.00c	0.88 ± 0.04c	4.175	0.004**
Foliar C:N	32.52 ± 2.57bc	25.67 ± 0.08c	47.77 ± 5.72a	40.16 ± 8.06ab	-4.870	< 0.001**
Foliar C:P	488.75 ± 28.65b	317.94 ± 27.23c	569.86 ± 10.52a	534.26 ± 22.50a	-4.286	0.003**
Foliar N:P	15.05 ± 0.48a	12.39 ± 1.03ab	12.06 ± 1.47b	13.77 ± 3.14ab	0.778	0.450
Foliar Mn (g kg^-1^)	1.00 ± 0.40a	1.77 ± 0.76a	1.72 ± 0.87a	0.88 ± 0.32a	0.235	0.818

EM species, ectomycorrhizal species; AM species, arbuscular mycorrhizal species; Foliar C, foliar carbon concentration; Foliar N, foliar nitrogen concentration; Foliar P, foliar phosphorus concentration; Foliar C:N, ratio of carbon to nitrogen in leaf; Foliar C:P, ratio of carbon to phosphorus in leaf; Foliar N:P, ratio of nitrogen to phosphorus in leaf; Foliar Mn, foliar manganese concentration. Different letter presents statistical difference among four species (P < 0.05); ** present statistical difference (P < 0.01) between EM and AM species.

### 3.4 The PLFAs and enzyme activity

**Figure 3:**
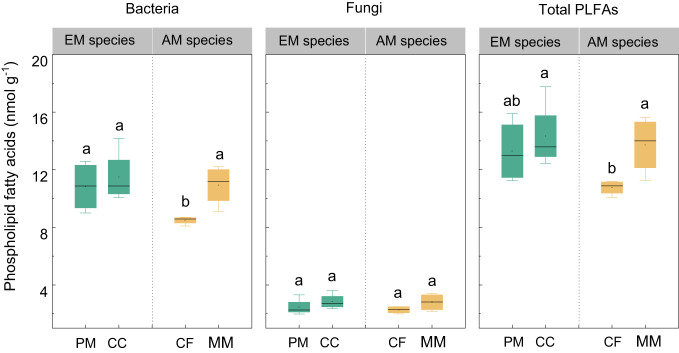
The phospholipid fatty acids (PLFAs) in *Pinus massoniana* (PM), *Castanopsis carlesii* (CC), Chinese fir (CF), and *Michelia macclurei* (MM) forest soils in subtropical China. EM species, ectomycorrhizal species; AM species, arbuscular mycorrhizal species. Different letter presents statistical difference among four species (*P* < 0.05).

**Figure 4:**
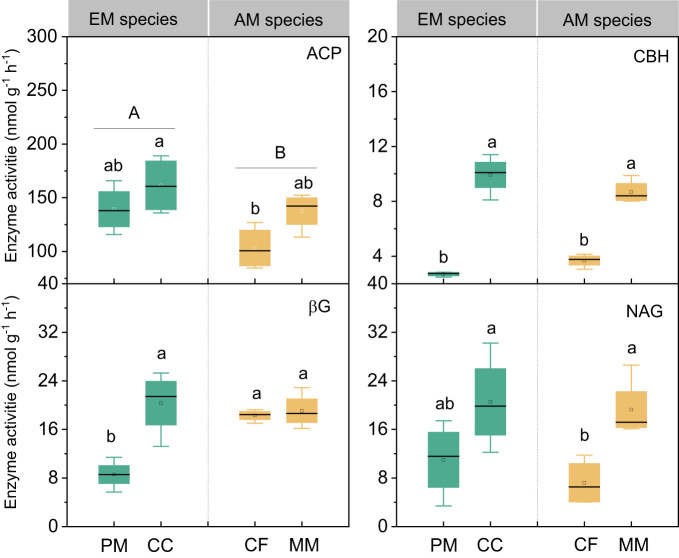
The enzymes activities in *Pinus massoniana* (PM), *Castanopsis carlesii* (CC), Chinese fir (CF), and *Michelia macclurei* (MM) forests in subtropical China. EM species, ectomycorrhizal species; AM species, arbuscular mycorrhizal species. ACP, acid phosphatase; CBH, cellobiohydrolase; NAG, β-N-acetylglucosaminidase; βG, b-1,4-glucosidase. Different lowercase letter presents statistical difference among four species (*P* < 0.05). Different uppercase letter presents statistical difference between EM and AM species (*P* < 0.05).

There were no statistically significant differences in bacterial, fungal, and total PLFAs between EM and AM species (*P* > 0.05, **Figure 3**). Similarly, no significant differences in these PLFAs were observed among the four forest soils (*P* > 0.05), except that the bacterial and total PLFAs in CF were lower than in other species (*P* < 0.05, **Figure 3**). Mycorrhizal type mainly altered the ACP activity, which was higher in EM species than in AM species soils (*P* < 0.05, **Figure 4**), whereas it did not affect the enzyme activity of CBH, βG, and NAG (*P* > 0.05).

### 3.5 Relationships between soil P fractions and soil properties, leaf nutrients, and microbial activity

**Figure 5:**
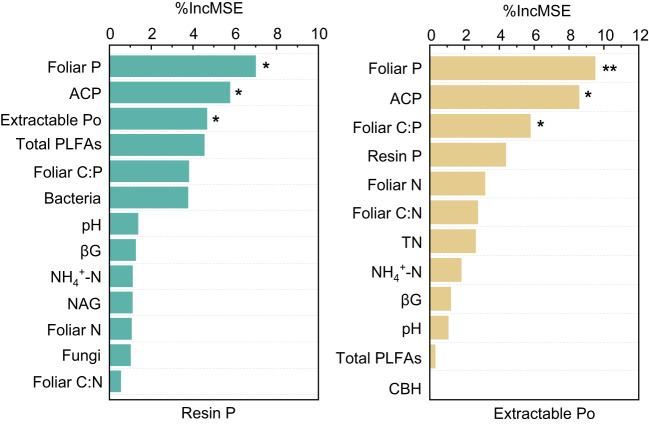
The dominant environmental predictors of available phosphorus (Resin P) and organic phosphorus (Extractable Po) in four subtropical forest soils. ACP, acid phosphatase; CBH, cellobiohydrolase; NAG, β-N-acetylglucosaminidase; βG, b-1,4-glucosidase; TN, total nitrogen. * and ** represent *P* < 0.05 and *P* < 0.01, respectively.

**Figure 6:**
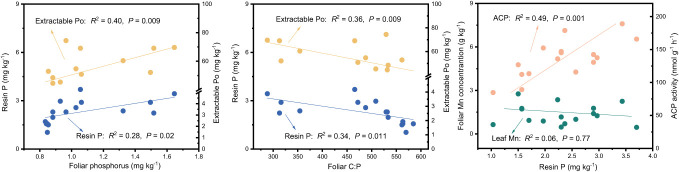
Relationships between soil phosphorus fractions and foliar phosphorus, ratio of carbon to phosphorus in leaf (Foliar C:P), foliar Mn and acid phosphatase (ACP) in four subtropical forest soils.

The results of the Random Forest model showed that foliar P, ACP, and extractable Po or foliar C:P were the dominant factors influencing resin P and extractable Po (*P* < 0.05), followed by bacteria, pH, foliar N, and NAG, and so on (**Figure 5**). Among these factors, resin P and extractable Po were positively correlated with foliar P (*P* < 0.02), but negatively correlated with foliar C:P (*P* < 0.01, **Figure 6**). In addition, a significant positive relationship was observed between resin P and ACP activity (*P* = 0.001), whereas no correlation was obtained between resin P and foliar Mn concentration (*P* = 0.77, **Figure 6**).

## 4 Discussion

### 4.1 Differences of soil P fractions between EM and AM species forests

**Figure 7:**
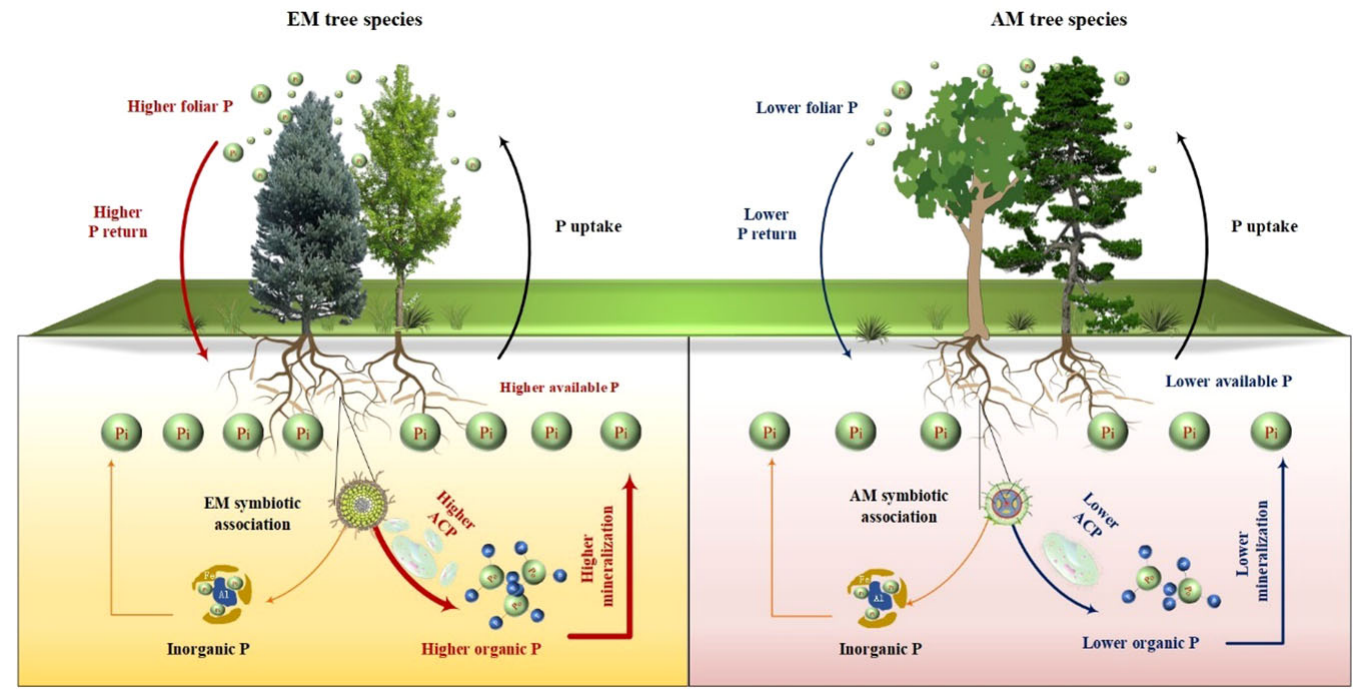
Conceptual graphic illustrating the influences of ectomycorrhizal (EM) and arbuscular mycorrhizal (AM) tree species on the availability and accumulation of soil P, as well as the underlying mechanisms. ACP, acid phosphatase.

Prior research has consistently recognized that EM species demonstrate a greater capacity to mobilize soil P, leading to increased soil P availability (Steidinger et al., 2014; Rosling et al., 2016; Fan et al., 2018). In line with these findings, our study also noted higher concentrations of available P (resin P and NaHCO_3_-Pi) in soils associated with EM species compared to AM species soils (*P* < 0.05, **Figure 1**). This may be associated with the differences in P utilization strategy between EM and AM species (Rosling et al., 2016; Fan et al., 2018). EM species have a great ability to mineralize organic P, as EM fungi can produce ACP. In contrast, AM tree species preferentially uptake inorganic P, as AM fungi lack the ability to synthesize ACP (Phillips et al., 2013; Rosling et al., 2016). However, a recent study in subtropical karst forests reported contrasting results, linking low P availability in EM species soils to reduced ACP activity (Yang et al., 2021). In contrast, another study found that P deficiencies stimulated the production of ACP activity in EM species (Meeds et al., 2021). Previous study have demonstrated that microbes-driven mineralization of organic P, through the production of ACP, serve as a key pathway for EM species to acquire soil P (Fan et al., 2018; Fan et al., 2019). In fact, this study also observed higher ACP activity in EM species compared to AM soils (*P* < 0.05, **Figure 4**) and identified a positive correlation between resin P and ACP activity (*P* = 0.001, **Figure 6**). This suggests that the higher P availability in EM species soil may be attributed to organic P mineralization (Fan et al., 2018; Fan et al., 2019). Importantly, we also found that changes in resin P were predominantly influenced by extractable organic P (**Figure 5**), underscoring the critical role of organic P in regulating available P in forest soils with different mycorrhizal types (**Figure 7**).

Furthermore, mycorrhizal type has an impact on organic P levels in forest soils (Koele et al., 2014; Rosling et al., 2016). For example, a study in temperate forests revealed that EM species led to lower organic P content (-28%) compared to AM species following an 80-year period of natural recovery in abandoned soils (Rosling et al., 2016). This difference may be attributed to the distinct P utilization strategies employed by tree species (Koele et al., 2014; Rosling et al., 2016). AM species predominantly rely on inorganic P, while EM species exhibit a greater ability to use organic P (Becquer et al., 2014; Plassard et al., 2019; Yu et al., 2023b). Consequently, organic P is more likely to accumulate in soils dominated by AM species, whereas it is more readily depleted in soils dominated by EM species. Besides, the elevated DON in MM soil compared to that in AM species (such as PM and CC) present an interesting finding. A likely biological explanation for this difference can be attributed to leaf protection mechanisms. Leaves of CC and PM species, with thick wax layers, are less prone to microbial decomposition. Conversely, the thinner wax layer on MM leaves facilitates more rapid decomposition, allowing foliar N being easily assimilated by microbes. This process results in a higher accumulation of organic N, such as DON, in MM soil. Additionally, the nutrient utilization strategies between EM and AM species show divergence (Phillips et al., 2013). EM species exhibit a substantial ability to utilize organic nutrients, AM specie primarily depend on inorganic nutrients and show a reduced capacity to break down organic matter (Phillips et al., 2013; Rosling et al., 2016). This disparity could explain the lower NO_3_^–^N content in EM species’ soils and the higher DON in soils with AM species.

As expected, mycorrhizal type did not trigger notable modifications in the concentration and proportion of unavailable P (e.g., extractable Pi and residual P) in the soils (*P* > 0.05, **Figures 1** and 2). This resistance to change could be attributed to the strong adsorption of these soil P by iron and aluminum components, as well as their association with internal surfaces of soil aggregates and secondary minerals (Hedley et al., 1982; Fan et al., 2021). These forms of P are not easily accessible to microbes or plant roots and are generally insensitive to environmental changes (Hedley et al., 1982; Tiessen and Moir, 1993; Zhang et al., 2020). This suggests that mycorrhizal type primarily influenced the dynamics of available P and organic P, with a lesser impact on the fractions of P strongly fixed in the soil.

Interestingly, the total P content was higher in soils associated with EM species compared to AM species (*P* < 0.05, **Figure 1**). This finding deviates from previous studies that did not detect significant alterations in total P among different tree species soils (Yang et al., 2021; Jiang et al., 2022). In general, P accumulation in the soil is primarily shaped by microorganisms and plants (Richardson and Simpson, 2011; Richardson et al., 2011; Yu et al., 2023a). In this study, no noteworthy distinction emerged in microbial biomass (e.g., bacterial and fungal PLFAs) and C and N hydrolysis enzymes (e.g., βG, CBH, and NAG) between soils with EM and AM species (*P* > 0.05, **Table S1**; **Figures 3**, **4**). One possible explanation is that soil properties, such as pH, SOC, TN, NH_4_^+^-N, showed no significant differences in EM and AM species soils (**Table 1**), indicating a similar capacity that provide C and N to microorganisms. As a result, no significant difference in synthesis of enzymes involved in the acquisition of C and N (Saiya-Cork et al., 2002). In contrast, ACP activity is higher in soils planted with EM species, suggesting a more pronounced difference in the impact of soil microbes on P cycling between EM and AM species (Rosling et al., 2016). This may due to the fact that microbes in subtropical soils are more limited by P rather than C and N (Plassard et al., 2019; Zhang et al., 2023). Given that EM species have a great ability to synthesize ACP, while AM fungi lack such ability (Phillips et al., 2013; Rosling et al., 2016). Therefore, ACP in EM species soil originates from both plant roots and EM fungi, potentially inducing higher ACP activity than in AM species soil. Altogether, the elevated levels of available P, extractable organic P, and total P in EM species soils compared to AM species imply that mycorrhizal type plays a pivotal role in soil P accumulation, with EM species exhibiting a greater propensity for P accumulation at the plot level (**Figure 7**). However, this finding is based on observations of two EM and AM tree species, evidences from more species are required to demonstrate the differential contribution of mycorrhizal type to P accrual in forest soils on a larger scale.

### 4.2 Dominant factors for P fractions accrual in EM and AM species forests soils

Plant-derived P, particularly foliar P, constitutes a major source of soil P, and its fluctuations might lead to variations in P accumulation and availability in soils (Vitousek, 1984; Sayer and Tanner, 2010; Gao et al., 2022). In this study, foliar P concentration was significantly higher in EM species in contrast to AM species (*P* < 0.05, **Figure 1**). These findings are consistent with the elevated levels of soil available P and organic P in EM species relative to AM species (**Figure 1**). Suggesting that the higher P availability in soil may be associated with greater plant-derived P input (Sayer and Tanner, 2010; Gao et al., 2022) (**Figure 7**). Moreover, the higher ACP activity in EM species soils is beneficial to hydrolyze foliar organic P, contributing to increases in available P in soil. This can be further supported by the findings from random forest analysis, which revealed that foliar P was the dominant factor regulating available P (resin P) and organic P (extractable Po) (**Figure 5**). Thus, it can be inferred that plant-mediated pathways governing P turnover processes may account for the observed variation in P content between EM and AM species soils (Richardson et al., 2009; Steidinger et al., 2014; Fan et al., 2019).

Besides, foliar nutrient stoichiometry also exhibited significant differences between EM and AM species, with AM species displaying higher foliar C:P ratios compared to EM species (**Table 1**, *P* < 0.01). This disparity may due to the elevated foliar P content in EM species. The higher foliar P content in EM species implies a great uptake of soil P by plants, potentially resulting in lower P accumulation in EM soils. However, both available P and organic P were higher in soils with EM species than AM soils (**Figure 1**). One possible explanation was the larger biomass of AM species, despite their lower foliar P. AM species (both CF and MM), known for their rapid growth (Chen et al., 2020; Wei et al., 2023), exhibited significantly higher tree height and diameter at breast height during the early stages of growth compared to EM species (PM and CC) (unpublished data), suggesting a higher biomass P in AM species than in EM species. These findings provide further evidence that the presence of AM species in the soil may lead to increased assimilation of P by plants, resulting in lower available P and organic P compared to soils with EM species (**Figure 7**).

The differences in foliar P between EM and AM species may suggest varying abilities to mediate the transformation among various P fractions, potentially affecting the proportion of different P fractions in total soil P (Rosling et al., 2016; Cheeke et al., 2017; Chang et al., 2022). However, the proportion of inorganic P (e.g., resin P, NaHCO_3_-Pi, and extractable Pi) to soil total P did not exhibit significant changes, whereas extractable Po and its proportion to total P were higher in soils dominated by EM species than in AM species (*P* < 0.05, **Figures 1**, **2**). This decrease in organic P may be attributed to its decomposition acting as the main P source for plant uptake (Becquer et al., 2014; Fan et al., 2018; Plassard et al., 2019). Two pieces of evidence support this: firstly, the dominant factor influencing resin P and organic P was found to be ACP (**Figure 5**); and secondly, the foliar Mn concentration, which serves as a proxy for plant capacity to mobilize inorganic P (Lambers et al., 2015; Yu et al., 2020; Lambers, 2022; Yu et al., 2023b), did not differ between EM and AM species (*P* > 0.05, **Table 2**), nor did it have a significant influence on available P and organic P (**Figures 5**, **6**). These findings collectively suggest that ACP-driven organic P mineralization is more vital than inorganic P mobilization for plant P uptake (Fan et al., 2018; Andrino et al., 2020), providing a reasonable explanation for the observed differences in organic P accumulation between EM and AM species (**Figure 7**).

Additionally, we found that interspecific differences also affect P accumulations in soils (Li M. et al., 2019). Such as the available P and organic P in soil hosting CF were lower than those in CC and PM soils (**Figure 1**). This is probably due to CF has a higher growth rate than other species, resulting in greater P accumulation in plant biomass and consequently reducing soil P levels. Therefore, it is crucial to consider the contribution of tree species when evaluating the influences of mycorrhizal associations on nutrient cycling and soil fertility in forest ecosystems.

## 5 Conclusions

This study demonstrated that EM species have a greater capacity for accumulating soil P (e.g., available P and organic P) compared to AM species at the plot level. One explanation is the higher return of plant-derived P (e.g., foliar P) to the soil during litter decomposition in EM species. Furthermore, organic P mineralization plays a more significant role than inorganic P desorption in influencing P availability in soils. These findings provide valuable insights into plant-soil interactions and emphasize the critical role mycorrhizal associations play in soil P dynamics. Further studies into changes in soil P associated with broader EM and AM species is essential to reinforce our findings and elucidate P cycling mechanism in forest ecosystems with limited P availability.

## Data availability statement

The original contributions presented in the study are included in the article/**Supplementary Material**, further inquiries can be directed to the corresponding author/s.

## Author contributions

PL: Data curation, Writing – original draft. LX: Investigation, Writing – review & editing. LY: Conceptualization, Data curation, Funding acquisition, Resources, Writing – review & editing. KY: Conceptualization, Funding acquisition, Supervision, Writing – review & editing. JP: Data curation, Funding acquisition, Methodology, Validation, Writing – review & editing.
