# Supplementary material for: Divergent soil P accrual in ectomycorrhizal and arbuscular mycorrhizal trees: insights from a common garden experiment in subtropical China
Source: Front Plant Sci. 2024 Feb 7;15:1333505. doi: 10.3389/fpls.2024.1333505 (PMC10879435; doi:10.3389/fpls.2024.1333505)
Supplement: Supplementary file 1 [file Table_1.docx]

| **Table S1** The phospholipid fatty acid (PLFA) in *Pinus massoniana* (PM), *Castanopsis carlesii* (CC), Chinese fir (CF), and *Michelia macclurei* (MM) forest soils in subtropical China | | | | | | |
| --- | --- | --- | --- | --- | --- | --- |
| PLFAs | EM species | | AM species | | *t* | P |
|  | PM | CC | CF | MM |  |  |
| GP (nmol g^-1^) | 5.51±1.01a | 5.90±1.02a | 4.21±0.24b | 5.32±0.45a | 2.254 | 0.041* |
| GN (nmol g^-1^) | 4.51±0.61ab | 4.71±0.67a | 3.60±0.22b | 4.75±1.07a | 1.098 | 0.291 |
| ACT (nmol g^-1^) | 0.81±0.15ab | 0.88±0.16a | 0.67±0.04b | 0.85±0.09a | 1.261 | 0.228 |
| AMF (nmol g^-1^) | 0.45±0.10a | 0.48±0.09a | 0.40±0.02a | 0.55±0.16a | -0.133 | 0.896 |
| EMF (nmol g^-1^) | 2.00±0.49a | 2.36±0.45a | 1.88±0.22a | 2.25±0.46a | 0.534 | 0.601 |
| Note: EM species, ectomycorrhizal species; AM species, arbuscular mycorrhizal species; GP, Gram-positive bacteria; GN, Gram-negative bacteria; ACT, Actinomycetes. AMF, Arbuscular mycorrhizal fungi; EMF, Ectomycorrhizal fungi. Different letter presents statistical difference among four species (*P* < 0.05); * present statistical difference (*P* < 0.05) between EM and AM species. | | | | | | |
